# Perceptions and Needs of Stakeholders Regarding MyPal Project’s Electronic Patient-Reported Outcome App: Cross-Sectional Qualitative Focus Group Study

**DOI:** 10.2196/57388

**Published:** 2025-08-13

**Authors:** Dimitrios Kyrou, Panos Bonotis, Christine Kakalou, Maria Vasilopoulou, Lydia Scarfò, Marcel Meyerheim, Annette Sander, Tomáš Arpáš, Eleni Kazantzaki, Christos Maramis, Christina Karamanidou

**Affiliations:** 1 Institute of Applied Biosciences Centre for Research and Technology-Hellas Thessaloniki Greece; 2 Strategic Research Program on Chronic lymphocytic leukemia, Division of Experimental Oncology Università Vita Salute San Raffaele Milano Italy; 3 Clinic of Pediatric Oncology and Hematology Faculty of Medicine Saarland University Hospital Homburg Germany; 4 Clinic for Paediatric Oncology and Haematology Hannover Medical School Hannover Germany; 5 Department of Internal Medicine – Hematology and Oncology University Hospital and Masaryk University Brno Czech Republic; 6 Department of Hematology University Hospital of Heraklion Heraklion Greece

**Keywords:** palliative care, eHealth systems, adult patients with cancer, health care professionals, focus group discussions

## Abstract

**Background:**

Palliative care is crucial for patients with life-threatening and serious diseases such as cancer, as it addresses their physical, psychosocial, and spiritual needs. Hematological malignancies significantly contribute to global cancer cases, impacting both older adults and children. To meet the increasing demand for palliative care, electronic patient-reported outcome (ePRO) interventions offer valuable insights into patient monitoring and treatment decision-making. The MyPal project developed a digital ePRO solution to improve palliative care by enabling structured symptom reporting and promoting physician-patient communication.

**Objective:**

This study aims to explore the perceptions, opinions, and needs of adult and pediatric patients with cancer, caregivers, and health care professionals (HCPs) regarding low-fidelity versions of the MyPal project’s digital solution, which is designed to improve palliative cancer care.

**Methods:**

A qualitative, cross-sectional study was conducted using 12 prepilot focus groups (FGs) across 4 European countries (Greece, Italy, Germany, and the Czech Republic) at participating hospitals and research centers. The FGs, held in person, included 61 participants, including 27 (44%) adult patients with chronic lymphocytic leukemia or myelodysplastic syndromes, 19 (31%) children with hematological malignancies or solid tumors and their parents, and 15 (25%) HCPs specializing in oncology and palliative care. A semistructured discussion guide, informed by vignettes and user personas, was used to facilitate discussions. Sessions were audio recorded, transcribed, and analyzed using thematic analysis to identify and extract themes and subthemes from the FG discussions.

**Results:**

Three main themes emerged from the FG discussions. The first theme, *improved care*, showcased the project’s potential to enhance health care through patient-reported measures by improving symptom monitoring, streamlining decision-making, and strengthening physician-patient communication. Patients and caregivers valued the ability to report symptoms remotely, reducing unnecessary hospital visits, while HCPs appreciated having structured patient data to guide treatment. The second theme, *digital communication framework*, revealed that while participants recognized the benefits of digital tools, they had concerns about data security, privacy, and clarity regarding communication protocols. Questions emerged about how and when HCPs would review and respond to patient-reported data. In the third theme, *applicability for use in health care*, participants emphasized the importance of the system’s ease of use, particularly for older patients and young children. Concerns were raised about the potential intrusiveness of the system, particularly regarding notification frequency and the impact on daily life. HCPs highlighted workload challenges, suggesting the need for a structured alert system to prioritize urgent cases.

**Conclusions:**

Our findings indicate that ePRO-based interventions such as MyPal can improve palliative care by facilitating communication and patient monitoring. However, addressing privacy concerns, optimizing usability for diverse populations, and ensuring seamless integration into clinical workflows are critical for successful adoption. Insights from this study will inform future development and optimization of eHealth interventions in palliative care.

## Introduction

### Background

Palliative care plays a vital role in the holistic treatment of patients with life-threatening and serious diseases such as cancer. Palliative care takes a multidisciplinary approach, focusing on preserving and enhancing the quality of life (QoL) for patients with life-threatening illnesses and their families [1]. Its primary aim is to prevent and alleviate distress through the early recognition, evaluation, and management of physical, psychological, emotional, and spiritual challenges. In addition, the World Health Organization emphasizes the importance of providing support systems to help patients maintain an active life throughout their disease trajectory [2]. As the global population ages, the demand for palliative care continues to grow due to the increasing prevalence of complex chronic diseases and progressive conditions, such as cancer [3,4]. To address these needs effectively, innovative interventions are essential.

Hematological malignancies (HMs) account for approximately 6.5% of all cancers worldwide and predominantly affect older individuals, with a median age of diagnosis of approximately 70 years [5-7]. Due to the chronic and often aggressive nature of these diseases, patients with HMs experience a significant symptom burden and diminished QoL compared to the general population [8]. This deterioration in QoL, coupled with the high prevalence of distressing symptoms, such as pain and fatigue, underscores the need for comprehensive palliative care approaches in this patient group. Furthermore, while cancer in children is relatively rare, it remains the leading cause of disease-related death in pediatric populations [9]. Among pediatric cancers, HMs are the most frequently diagnosed and profoundly impact not only young patients but also their families and caregivers [10]. Given the distinct challenges faced by pediatric patients, palliative care plays a critical role in symptom management and improving their overall well-being and QoL throughout their disease trajectory [11].

With the growing demand for palliative care and the limited number of specialized health care professionals (HCPs), it becomes critical to identify methods to optimally use available resources [12]. Palliative care is a resource for anyone living with a serious illness. It extends beyond end-of-life support, integrating early into the disease trajectory alongside active treatment to improve symptom management, psychological well-being, and overall QoL [12]. One promising approach is the use of electronic patient-reported outcomes (ePROs), which have been integrated into various palliative care interventions for patient monitoring, providing reliable data and improving the quality of care delivered to patients with cancer [13]. eHealth tools and applications offer promising solutions for enhancing patient monitoring and care coordination. However, their adoption is hindered by barriers, such as low engagement among older adults and disease-specific usability factors [14]. Participatory design [15], which actively involves patients in developing eHealth tools [16], resources [17], or systems [18], has shown potential to enhance user acceptability, usability, and the feasibility of interventions in palliative care [19]. While usability concerns exist for older adults, children face distinct engagement challenges, as demonstrated in the MyPal4Kids study [20]. Younger children often benefit from engaging, gamified designs, whereas older children may find such approaches too simplistic, leading to disengagement. Tailoring eHealth tools to the developmental and cognitive needs of different age groups is essential to optimize usability, engagement, and effectiveness.

### MyPal Project

Building on these principles, the MyPal project is a collaborative Horizon 2020 research project, funded by the European Commission, aiming to use eHealth technologies in order to support patients with cancer and HCPs. The main goal of MyPal is to develop and clinically assess new ePRO-based interventions for the palliation of patients with cancer in order to improve their QoL [21]. The project, through the MyPal digital solution, targets both adults with chronic HMs via an ePRO-enabled mobile app and pediatric patients with leukemia or solid tumors via a serious game tablet app, along with their caregivers. The digital solution also comprises a web-based HCP portal. The pediatric patient’s app is characterized as a “serious game” because it serves a purpose beyond entertainment, aiming to engage pediatric patients in their care journey by improving health literacy, encouraging symptom reporting, and fostering adherence to treatment protocols. The MyPal project has committed to both adopting a patient-centered approach and adapting technology in order to cater to fundamentally different profiles of patients of different age groups as well as levels of digital and health literacy. Therefore, during the MyPal intervention design and protocol development, not only the context of health care provision, that is, current clinical practice for patients with cancer, their interaction with HCPs, and provision of palliative care but also users’ personal needs were considered.

### Objective

The purpose of this study is to explore the perceptions, opinions, and needs of HCPs, adult patients with cancer, and caregivers of pediatric patients with cancer regarding the MyPal digital solution, a palliative care eHealth solution. Specifically, we aim to understand users’ views on system functionalities, benefits, challenges, and potential improvements.

## Methods

**Figure 1:**
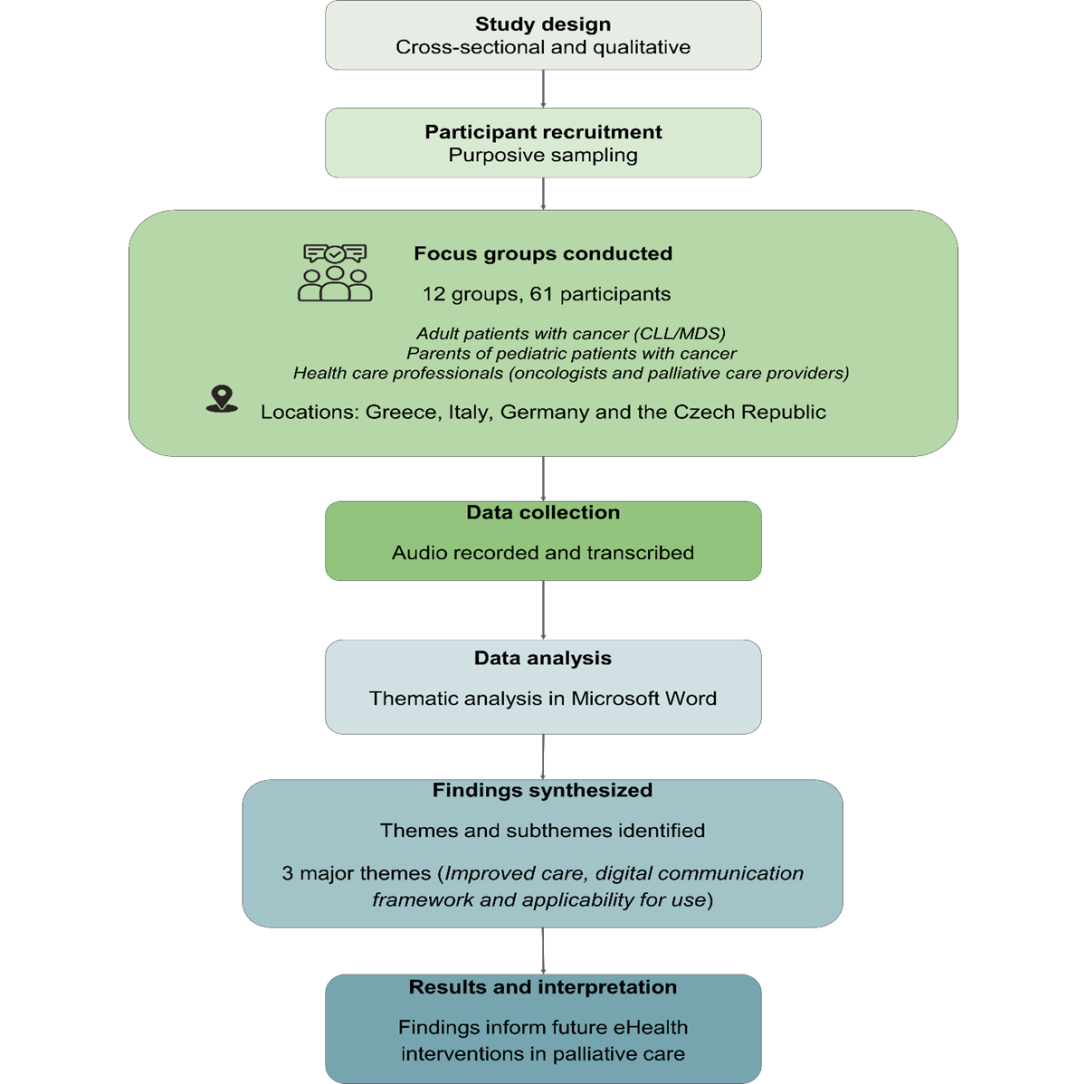
Study flowchart depicting the overview of the study process from design and participant recruitment to focus groups, data collection, analysis, and key findings. CLL: chronic lymphocytic leukemia; MDS: myelodysplastic syndrome.

The chronological steps of the study process are presented in Figure 1.

### Study Design

This study used a cross-sectional qualitative methodology, using focus group (FG) discussions to explore the perceptions of patients, caregivers, and HCPs regarding MyPal, an ePRO-based digital solution for palliative care (NCT04370457) [8]. This study was conducted in 5 clinical sites across 4 European countries (Greece, Italy, Germany, and the Czech Republic).

It involved 3 participant groups: (1) adult patients with chronic lymphocytic leukemia (CLL) or myelodysplastic syndromes (MDSs), (2) children with HMs or solid tumors and their parents, and (3) HCPs working in participating clinical sites.

Each FG was moderated by an interdisciplinary team, comprising professionals experienced in health care (LS: female physician; MM: male PhD candidate; AS: female physician; TA: male physician; EK: female PhD candidate; and C Karamanidou: female PhD graduate) and psychosocial work at each clinical site. The moderators received previous training and possessed experience in conducting and leading FGs within the fields of health care and health psychology and had no previous relationship with the participants, who were solely informed of their professional roles and qualifications. The interviews were conducted in each country’s native language (Greek, Italian, German, and Czech). They took place in an assigned meeting room within each of the 5 hospitals (Università Vita-Salute San Raffaele [USR; Vita-Salute San Raffaele University], Universität des Saarlandes [USAAR; Saarland University], Medizinische Hochschule Hannover [MHH; Hannover Medical School], Fakultní nemocnice Brno [BRNO; University Hospital Brno], Panepistimiako Geniko Nosokomeio Irakleiou [PAGNI; University General Hospital of Heraklion]) and in one research center (Centre for Research and Technology Hellas [CERTH]) where participants’ privacy was protected.

### Participant Recruitment

Recruitment was carried out by the principal investigators from each clinical site. Invitations were extended in person, via email, or by telephone to patients, HCPs, and parents of young patients. Specifically, participants for each participant group were recruited if they fulfilled the following criteria: (1) patients with CLL or MDS, (2) HCPs who worked in palliative care units or oncology units, and (3) parents of young patients (with or without their children) with solid tumors or HMs. The participants were recruited through the hospital units where they worked or were being looked after. This study used a mixed sampling method to recruit participants. HCPs were recruited using purposive sampling, targeting those with experience in palliative care or oncology, while patients with CLL or MDS and parents of young patients were recruited via random sampling. For the purposes of participant recruitment, a screening paragraph was developed to help HCPs from each participating site recruit participants in a standardized way:

Within the context of a project called MyPal funded by the European Commission we are organizing a number of focus groups discussions. We are interested in developing a digital health system for cancer patients [or for HCPs looking after cancer patients] and we would very much appreciate your input as our design should ideally correspond to the needs of patients [or HCPs] like yourself. Let us know whether you would be interested in taking part.

### Development of FG Content

#### Overview

**Figure 2:**
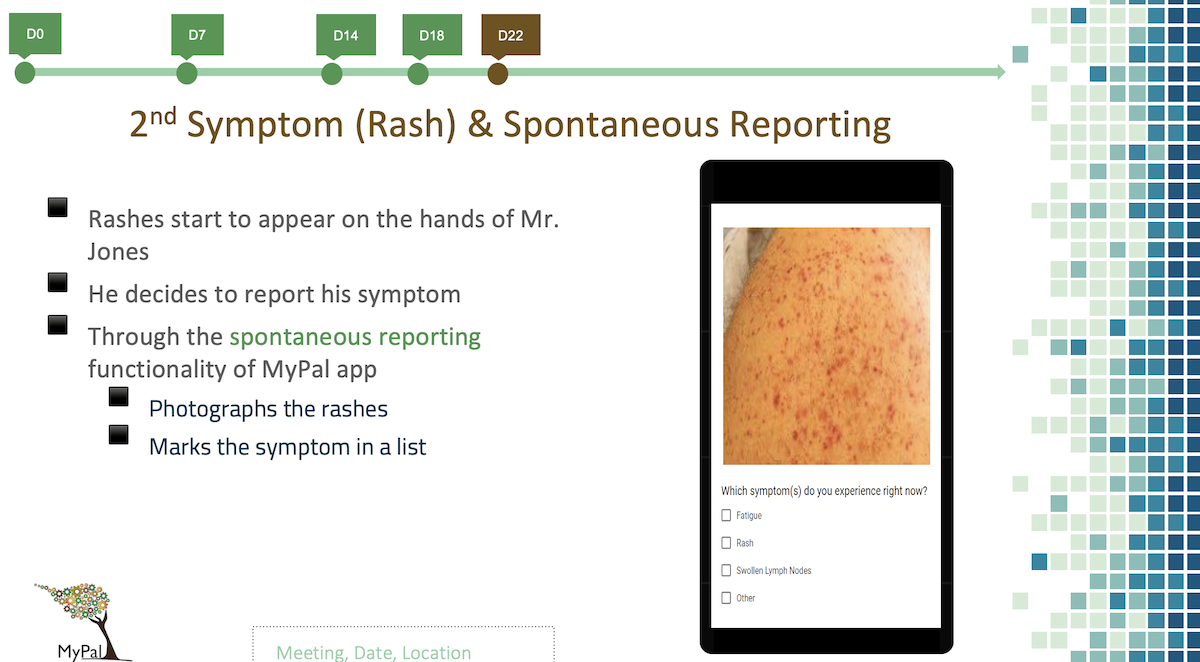
Vignette for adult patient.

A series of vignettes was developed by a female (C Kakalou) and a male (CM) software engineer, with expertise in eHealth and participatory design techniques, and a female health psychologist (C Karamanidou). These vignettes presented more than 1 imaginary end users (called personas) of the system and targeted each participant group (Multimedia Appendix 1). Each vignette introduced a persona and illustrated how it interacted with the MyPal app and all its components from enrollment to the clinical trial up until its end (Figure 2).

On the basis of the vignettes, discussion guides were created in order to assist the elicitation of participants’ perceptions and judgments in a semistructured way (Multimedia Appendix 2). The discussion guides included showing the user a scenario divided into episodes and then asking relevant questions to the participants. Importantly, all key materials (the discussion guide, the user scenario, and the consent form) were provided to FG participants in English.

#### Training Workshop

Aiming at maximizing consistency in the conduct of FGs and the analysis of the data across every clinical site, the Institute of Applied Biosciences (INAB) at the CERTH developed a 2-hour training workshop, which was delivered online to all MyPal participating clinical sites and partners (Multimedia Appendix 3). The workshop was recorded so that those who could not be present could view it at their own time.

The workshop was held after the completion of the first 2 FGs in Greece. The aim of the workshop was as follows: (1) to present the rationale behind interacting with potential users to elicit needs and preferences via FG discussions and (2) to present the process that must be followed to ensure consistency among sites.

FG facilitators were given a list of necessary materials (ie, recorder) and conditions that had to be met (ie, private room) in order to successfully conduct a FG on the premises of their organization (ie, hospital ward). Particular emphasis was placed on the recruitment of FG participants and the role of the moderator, both of which were discussed at length as they could have an impact on the integrity of the methodological design. Examples of each were presented and discussed on the context. For example, certain questions from the discussion guide were phrased as open-ended questions to avoid judgment, etc.

Following this, managing the FG discussion itself was discussed with the workshop attendants. Specifically, with regards to group dynamics, attendants were advised to pay attention to nonverbal language; manage “airtime” among members; as well as use techniques to invite, reflect, or summarize.

Finally, 3 examples from the FGs conducted in Thessaloniki were presented. Attendants had the opportunity to comment on the role of the moderator and their choice of intervention at each instance as well as the sequence or the manner the questions were posed. Furthermore, attendants were able to observe from a bird’s eye view the stages of the process the group undergoes after the introduction of a new item on the agenda by the moderator, namely exploration, unison, and solution. Key references on the conduct of FGs [22] and a step-by-step thematic analysis guide [23] were provided as supplementary material.

### Data Collection

The FGs initially featured a presentation of the appropriate vignette, followed by a specific discussion on this vignette based on the discussion guide. At the beginning of each session, the aim of the MyPal project was introduced, followed by an explanation of the session’s structure.

None of the participants refused to participate or dropped out during or after the FGs. All FG discussions were conducted exclusively with the researchers and participants present. Each FG was audio recorded and lasted approximately 2 hours. Data collection was considered complete once participants’ accounts had been analyzed, and the resulting conceptual structure was determined to be sufficiently rich and detailed to address the research questions and meet the analytic objectives of the study. During the FGs, no field notes were taken, transcripts were not returned to participants, and no repeat FGs were conducted as the spontaneous and instinctive nature of participants’ responses was considered essential for capturing the authenticity of their experiences. Transcription and translation followed a systematic approach to preserve accuracy and consistency. Initially, all discussions were transcribed in the original language of the FG (Greek, Italian, German, or Czech). Each research team was responsible for translating the transcripts and the participants’ quotes into English, ensuring that the original meaning and context were accurately conveyed. To maintain consistency across sites, translations were reviewed within each team, safeguarding the fidelity of participants’ narratives in the final dataset.

### Data Analysis

The qualitative data were analyzed using thematic analysis, which is an established method of management and analysis of qualitative data in applied health research [24] and usability for mobile apps [25]. The initial inductive coding was carried out independently by the research team of each participating site. Subsequently, the coded data were consolidated to ensure consistency and facilitate cross-site analysis. The aggregation and the final analysis for this study were conducted by a male clinical psychologist (DK) with previous experience in thematic analysis using an inductive approach. The analysis was performed in Microsoft Word.

Ensuring uniformity of the FG analysis in the different participating sites was the primary concern of the research team. To address this challenge, the clinical psychologist consolidated the codes generated from different countries, and thematic analysis was applied to identify central themes and subthemes. This process facilitated the generation of a cohesive and integrative analytic outcome that adequately accounted for the diversity present within the dataset. Among the main reasons behind choosing this method of analysis were its suitability for exploring and identifying patterns in qualitative data, particularly participant perceptions, which aligned with the aim of this study; the multidisciplinary research teams involved, which facilitated a comprehensive interpretation of the data; the sample composition, which included clinical, patient, and lay representations, requiring a method capable of capturing multifaceted views regarding specific features of the MyPal digital solution; and the complexity of the dataset, which was generated via the collection of data in 4 different countries and languages, requiring a flexible and robust analytic framework. According to the guidelines by Braun and Clarke [23,26,27], the steps that were followed were as follows:

1. Familiarization with the qualitative data was carried out through listening to the FG discussions.
2. Inductive coding of the FG data to generate preliminary concepts was conducted. These codes were developed at a national level. Examples of preliminary codes generated were “The physician has the chance to evaluate signs and symptoms of which the patient is not aware” and “It is easy, to the point, immediate.”
3. Inductive codes were sent to the INAB at the CERTH, and the final stages of analysis were performed on the aggregated data.
4. The initial codes were clustered by the clinical psychologist into overarching categories that reflected conceptual patterns across the data (subthemes and themes).
5. The external heterogeneity and internal homogeneity of the developed categories were reviewed in a multidisciplinary research team (health and clinical psychologists [C Karamanidou and DK], eHealth [CM and C Kakalou], and usability experts [PB]) and refined until consensus was reached.

While the thematic analysis was conducted on aggregated data from all participating countries, initial coding was performed at the national level to account for local context and language. During the consolidation phase, the clinical psychologist leading the analysis reviewed the codes from each country to identify both shared and divergent patterns. Although some nuances specific to cultural or health care contexts emerged during coding, no substantial thematic differences were identified that warranted separate country-level themes. This decision was further validated through discussions with the multidisciplinary research team, ensuring that the core concepts identified were consistent across sites. Thus, aggregation was deemed appropriate to provide a comprehensive overview of stakeholder perceptions. Nonetheless, potential differences in system adoption and perception across countries are expected to emerge more clearly during the evaluation phase of the MyPal project.

### Ethical Considerations

The integration of ePROs via mobile apps has raised ethical concerns regarding inclusion criteria, information provided to participants, free and voluntary consent, and respect for their autonomy. These have been carefully addressed by a multidisciplinary team. Data processing, dissemination, and the use of the study findings will take place in full compliance with European Union data protection law. A participatory design was adopted in the development of the digital solution, involving FGs and discussions with patients to identify needs and preferences.

Before the interviews, participants were informed about the study’s objectives. They were required to sign a consent form outlining the measures taken to protect their confidentiality; the processes for anonymizing, storing, and managing the data; and their rights to refrain from answering specific questions or withdraw from the study at any time. Ethics approval for the study was obtained from the research ethics committee of the INAB at the CERTH and the research ethics committee of San Raffaele (8/2020), Thessaloniki “George Papanikolaou” Hospital (849), Karolinska Institutet (20.10.2020), University General Hospital of Heraklion (07/15.4.2020), and University Hospital of Brno (01-120220/EK). The study adhered to the principles in the Declaration of Helsinki and complied with the European General Data Protection Regulation. Special attention was given to safeguarding participants’ identities and maintaining the confidentiality of their narratives. Because qualitative data often contain detailed personal experiences and sensitive information, all transcripts were deidentified, replacing real names with pseudonyms or participant codes. Any contextual details that could reveal participants’ identities were removed or generalized to ensure anonymity. Access to raw transcripts was strictly limited to the research team members responsible for data analysis, and all files were securely stored in encrypted databases. Participation in the study was entirely voluntary, and no form of compensation was provided.

## Results

### Overview

A total of 61 participants took part in 12 FGs (3-8 participants per group), which were conducted across 5 clinical sites in 4 European countries (Greece, Italy, Germany, and the Czech Republic; Table 1). Participants included adult patients with CLL or MDS, children with HMs or solid tumors and their parents, and HCPs specializing in palliative care or oncology. Among them, 27 (44%) were adult patients (men: n=15, 56%; women: n=12, 44%; age: mean 61, SD 11), most had received treatment (n=25, 93%), with several in relapse or partial remission, while others were treatment naive (n=2, 7%). Detailed demographic and clinical characteristics of all participant groups are summarized in Table 2, including age, sex, and professional roles where applicable.

**Table 1:** Summary of the focus groups (FGs) by country.

Country, clinical site/FG number		Adult patient FGs (n=27), n (%)^a^	Parent and child FGs (n=19), n (%)^b^	HCP^c^ FGs (n=15), n (%)^d^
**Greece**				
	CERTH^e^ FG1	4 (15)	—^f^	—
	CERTH FG2	5 (19)	—	—
	PAGNI FG1	5 (19)	—	5 (33)
**Italy**				
	USR^h^ FG1	4 (15)	—	—
**Germany**				
	MHH^i^ FG1	—	4 (21)	6 (40)
	USAAR^j^ FG1	—	7 (37)	4 (27)
**The Czech Republic**				
	BRNO^k^ FG1	3 (11)	8 (42)	—
	BRNO FG2	6 (22)	—	—

^a^n=6 adult patient FGs.

^b^n=3 parent and child FGs.

^c^HCP: health care professional.

^d^n=3 HCP FGs.

^e^CERTH: Centre for Research and Technology Hellas.

^f^Not applicable.

^g^PAGNI: Panepistimiako Geniko Nosokomeio Irakleiou.

^h^USR: Università Vita-Salute San Raffaele.

^i^MHH: Medizinische Hochschule Hannover.

^j^USAAR: Universität des Saarlandes.

^k^BRNO: Fakultní nemocnice Brno.

**Table 2:** Participant characteristics.

Characteristics		Adult patient FG^a^ (n=27)	Parent and child FG (n=19)			HCP^b^ FG (n=15)
			Children (n=10)	Parents (n=9)		
**Sex, n (%)**						
	Female	12 (44)	2 (20)	6 (67)	10 (67)	
	Male	15 (56)	8 (80)	3 (33)	5 (33)	
Age (y), mean (SD; range)		61 (11; 39-83)	13.50 (1.75; 10-17)	43 (3; 37-49)	42.50 (3.75; 35-50)	
**HCPs’ specialties, n (%)**						
	Oncologists	—^c^	—	—	9 (60)	
	Nurses	—	—	—	3 (20)	
	Other HCPs	—	—	—	3 (20)	

^a^FG: focus group.

^b^HCP: health care professional.

^c^Not applicable.

Of the 61 participants, the parent and child FG included 10 (16%) children (boys: n=8, 80%; girls: n=2, 20%; age: mean 13.50, SD 1.75 y) diagnosed with leukemias, lymphoma, sarcoma, or epidermolysis bullosa (all undergoing active treatment) and 9 (15%) parents (mothers: n=6, 67%; fathers: n=3, 33%; age: mean 43, SD 3 y). In addition, 15 (25%) HCPs (men: n=5, 33%; women: n=10, 67%; age: mean 42.50 y; SD: 3.75) participated, including oncologists (n=9, 60%), nurses (n=3, 20%), and other HCPs (n=3, 20%), such as a physiotherapist, a social worker, and a psychotherapist, with experience in oncology settings, pediatric hemato-oncology or palliative care. The FGs varied in size and were structured to capture diverse perspectives on the MyPal digital solution.

**Figure 3:**
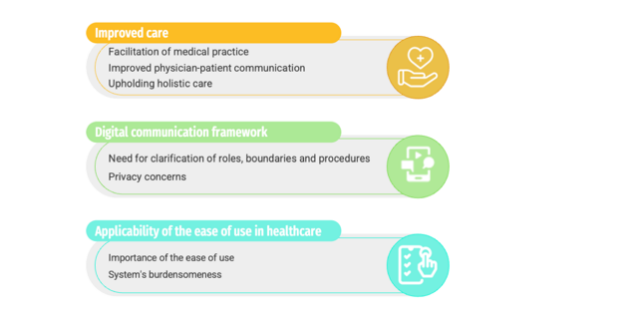
Main themes and subthemes identified through the thematic analysis methodology.

Three themes and 7 subthemes (Figure 3) were developed from the FG data. The first theme (improved care) portrayed participants’ perceptions and opinions regarding MyPal’s potential to improve health care using patient-reported measures and outcomes, which can both enhance monitoring and decision-making, improve physician-patient communication, as well as highlight areas of intervention (eg, psychological distress), which can easily go unnoticed in clinical practice. The second theme (digital communication framework) highlighted participants’ need for more explicit clarification of the boundaries, roles, and procedures regarding MyPal’s digital communication framework and their concerns regarding the privacy of their personal data. The third theme (applicability of use in health care) described the importance of the system’s ease of use, presented participants’ feedback regarding MyPal’s burdensomeness, and presented participants’ ideas on how to enhance its acceptability for health care use.

Themes and subthemes are presented in more detail subsequently, along with relevant quotes from the FGs, which further support and illustrate the presented concepts. The quotes’ descriptions include the participant group, the clinical site, and the specific functionality of the MyPal digital solution on which participants commented, that is, patient-reported outcomes, smart bracelet, personalization, general judgment, facial recognition and voice recognition, and game (for the MyPal-CHILD study only).

### Improved Care

#### Facilitation of Medical Practice

Patients, parents, and HCPs reported that MyPal could promote an easier and more efficient way of symptom monitoring and reporting, especially through the patient-reported measures. Emphasis was placed on the convenience of reporting symptoms via the app, without having to visit the hospital or call the physician:

I think it helps, because patients don’t want to visit the doctor every month, so reporting from home will be easier and less stressful.

General judgment; adult patient FG, parent and child FG, and HCP FG; USAAR

Patients highlighted that by self-reporting their symptoms, they would become better at self-monitoring and enable their health care team to keep better track of their condition. Specific functionalities, such as photo reporting and the smart bracelet, were emphasized as a means for more accurate reporting:

The patient may not describe or may not be able to accurately describe a symptom, for example, a rash. A photo is far more explanatory.

Patient reported outcomes; adult patient FG; CERTH

It is very useful, I’ll be constantly monitored.

Smart bracelet; adult patients; USR

HCPs pointed out the value of having immediate access to accurate patient data that can help in the documentation of patients’ states (eg, symptoms and side effects). In addition, some mentioned that the collected data could help with more efficient decision-making in medical practice:

This system could help with some decisions, like how often to monitor a patient.

Smart bracelet; HCPs; PAGNI

On the contrary, some HCPs mentioned that they would be hesitant to shape their decisions based on the collected data and becoming informed about patients’ symptoms through the app and not by physically examining the patient would cause them to feel uncertain and worried regarding the symptoms’ importance:

For me, as a doctor, remote monitoring would not be to my liking. My experience has shown that if you do not see the patient, you can be sure what he has, you cannot feel safe with the recordings.

Smart bracelet; HCPs; PAGNI

#### Improved Physician-Patient Communication

Every group of participants highlighted the fact that MyPal could improve physician-patient communication through the fast and direct reporting of symptoms. Of note is that both patients and HCPs among many different FGs mentioned that MyPal could alleviate patients’ fear of burdening their physicians and enable them to report more symptoms without hesitating:

It’s helpful for patients who hesitate to disturb their physician, because they don’t know whether their symptoms are important.

Patient reported outcomes; HCPs; CERTH

In one FG with HCPs, the value of MyPal questionnaires that would be used to personalize health care to each patient’s profile was more than emphasized. HCPs described how by getting to know each patient more through MyPal, they could tailor their communication to their specific needs:

By using MyPal you can understand how a patient might be feeling when sometimes they become demanding in our appointments. Knowing things about them and their experiences might enable us to respond to their questions more promptly. This might address their issues, even remotely.

Personalization; HCPs; PAGNI

In addition, some HCPs mentioned that the discussion guide and communication training functionalities of the app could be helpful in better equipping them to respond to their patients’ needs. However, many participants noted their preference for more personal contact via the phone or in person and felt that communicating only via MyPal could be depersonalizing:

I would prefer more calls and more personal contact.

General judgment; HCPs; USAAR

Children have their favorite doctors and they like contacting them directly. But they do not exclude the contact with the app.

Communication; parents and children; BRNO

#### Upholding Holistic Care

HCPs, patients, and parents highlighted MyPal’s potential to support patients not only regarding the physical aspects of their condition but also the emotional and psychological aspects. According to participants, frequent reporting through the app could mitigate the anxiety about the disease and make patients feel secure and reassured without having to contact their physician. In addition, reporting data regarding patients’ emotional challenges could promote holistic health care and improve patients’ QoL:

It would provide important information regarding how the patient is feeling. This might help because if we know that one is not fine, next time we could spend more time with them. If we notice that one is depressed, we could refer them to a psychologist. You can’t treat someone who is depressed and miserable. This isn’t good, even for their medical treatment. So, when you have access to this information, you can use it as you wish.

Patient-reported outcomes; HCPs; PAGNI

It might be helpful in addressing aspects that would normally not be addressed without data from MyPaL.

Communication; HCPs; MHH

Patients, parents, and HCPs highlighted that the app could provide patients with a sense that they are not alone and their health care team is there for them and takes note of their everyday struggles:

I think that this is psychotherapeutic for the patient, at times when they have no one to talk to. It mostly helps the patient, not us.

Patient-reported outcomes; HCPs; PAGNI

In addition, some patients mentioned that the personalized education functionalities of the app could be a relief and save them from the stress of searching on the internet:

It is reassuring. This app is personalized and tailored to my needs. I would be more relaxed, less anxious.

Personalization; patients; USR

Concerning children with HMs, their parents commented that the serious game could act as a distraction and attenuate their pain as well as become a channel for fun and communication with peers. In addition, some parents and HCPs commented that the app might motivate children to be more active physically and empower them to better address emerging issues:

It would help physically and psychologically, by motivating them to stay active regarding their health issue and deal with it in better ways.

General judgment; HCPs; PAGNI

Finally, patients and HCPs in Greece highlighted the need for cooperation with mental health specialists in order to provide support for the psychological issues that would be reported by patients through the app:

It would provide us with valuable insights into what the patient is experiencing [...] If we notice that they are depressed, we could refer them to a collaborating psychologist. You can’t treat a sick person who is depressed and unhappy.

Facial recognition; HCPs; PAGNI

Need to speak immediately with a specialist, perhaps even a mental health professional.

Facial recognition; patients; PAGNI

### Digital Communication Framework

#### Need for Clarification of Roles, Boundaries, and Procedures

The description of MyPal raised several questions and concerns for participants. Patients and HCPs expressed their confusion regarding the use of telephone calls while using MyPal, that is, in which instances they could contact their physician via phone and in which instances they should report their symptoms via the MyPal mobile app. In addition, patients expressed the need to know who will be the HCP that receives their reported information and how often they will be viewing the reported symptoms and reply:

Is it possible to get a response at night hours, when something happens?

Communication; parents; BRNO

If I experience something at 4:00 a.m. and record it, when will the doctor be able to review it? Will they be notified and check it in the morning?

Communication; patients; PAGNI

Furthermore, several patients expressed the need to receive feedback from their HCPs after submitting information to the app. Patients would like to receive immediate feedback tailored to their unique reported symptoms instead of automatic responses. Along these lines, parents mentioned the need to be able to receive confirmation that things are under control when their children submit information about their symptoms. In 1 FG, patients also expressed the need for personalized medical advice:

For instance, if you have high blood pressure, use less salt, or consider walking more.

General judgment; patients; PAGNI

By contrast, HCPs made the point that patients should be informed explicitly that the MyPal digital solution is not for emergency situations and that they should make a call or go to the hospital in such cases.

Finally, both patients and HCPs raised concerns regarding the appropriate interpretation and clinical value of the data captured by the face recognition and the smart bracelet utilities of the app:

If I have a fight with my husband and my facial expression is changing, this is not due to the disease.

Facial recognition; patients; USR

I would trust more seeing the patients’ face myself, rather than leaving the system to estimate.

Facial recognition; HCPs; USAAR

We cannot know what the measurements reflect.

Smart bracelet; HCPs; USAAR

#### Privacy Concerns

Every group of participants perceived that the smart bracelet and face and voice recognition utilities would invade their privacy, along with personal questions that could potentially be included in the MyPal questionnaires for personalization purposes:

One could feel as being constantly watched, like in George Orwell’s book, a sort of Big Brother. Too much personalization could be misunderstood.

Personalization; patients; USR

They also expressed their concerns regarding the security of their personal data and highlighted the need for safeguarding their confidentiality. Some mentioned that the face and voice recognition could be acceptable only under the condition that the patients could choose when to enable it:

Another question involves the sensors, microphone and camera. If one goes to the toilet, will the camera film that?

Facial recognition; parents and children; USAAR

If I had control over the timing of the recordings...yes.

Facial recognition; patients; CERTH

### Applicability for Use in Health Care

#### Importance of the Ease of Use

Some patients and parents wondered whether patients with CLL, who are generally older, will have the necessary technical skills to be able to use the MyPal digital solution:

The main criticism on the tool is that patients with CLL are generally elderly and they are not very familiar with this technology. If I think of my mother, who is 84 years old, I see this issue.

General judgment; patients; USR

The same issue was raised for children and people who do not have a smartphone. By contrast, other patients viewed the MyPal digital solution as an opportunity to become familiar with technology:

Technologies are the future and it is necessary to move with the times when it comes to health.

General judgment; patients; BRNO

Finally, patients showed appreciation for features that could facilitate the ease of use even more, such as conversational agents (eg, chatbot) or voice-activated functionalities.

#### Concerns About the System’s Intrusiveness and Burdensomeness

Participants raised concerns regarding the intrusiveness of the MyPal digital solution in many FGs. Frequent notifications for the completion of PRO measures were perceived as a constant reminder of CLL, which would burden patients and interfere with their everyday life, both practically and emotionally:

They would remind the patient that he is ill even at times when he may not be conscious of it.

Personalization; patients; CERTH

Participants suggested that receiving notifications and having to fill PRO measures should be sparse and personalized to their preferences to not tire them out and make them lose their motivation to use the MyPal digital solution. The intervals suggested by the participants varied. For some, weekly reporting was considered acceptable, while for others, monthly reporting was considered more acceptable. Wearing a smart bracelet was considered a burden by some, while others believed it was completely acceptable.

In addition, a few participants expressed their concern regarding addiction to the app and spending too much time on the mobile phone, especially regarding young children.

Of note were HCPs’ concerns regarding the time they would have to spend using the MyPal digital solution. With few exceptions, having to look at the extra information that MyPal added to clinical practice was perceived as an extra workload. In addition, having to respond to patients, read the spontaneously reported messages, document their progress according to PROs, and fill the patient’s search engine with appropriate educational material was perceived as highly burdensome:

How will MyPal be added to the regular business day of physicians who already have packed schedules for example if there are incoming SMSs because of parental reports? We already have a good system, perhaps in other countries this may be helpful, but how will it be incorporated here? We ask parents to call at the ward in case of problems, how will the use of the app be integrated? It will be difficult, I don’t know how it can be integrated.

Patient reported outcomes; HCP; MHH

In addition, HCPs highlighted that they would not want to feel that they are constantly on a call or receive notifications during their free time:

If I am not at work, I wouldn’t want to receive a notification that a patient is not well.

General judgment; HCP; USAAR

Along these lines, HCPs suggested developing a smart notification system, which could incorporate patients’ data and notify physicians only under certain conditions. Alternatively, some suggested the development of a traffic light system, which would categorize patients’ reported data according to their severity. This would allow HCPs to quickly track important patient data without having to review the whole dataset.

If there is an alert for data [red light and green light] it could take only a few minutes.

Patient reported outcomes; HCPs; USAAR

## Discussion

### Principal Findings

This study explored the perceptions of patients, caregivers, and HCPs across 12 FGs in 5 clinical sites spanning 4 European countries regarding MyPal, an ePRO-based digital palliative care solution. Three major themes emerged highlighting both the potential benefits and challenges of integrating digital tools into palliative care: (1) *improved care*, emphasizing MyPal’s potential to enhance symptom monitoring and physician-patient communication; (2) *digital communication framework*, highlighting privacy concerns and the balance between digital and direct interactions; and (3) *applicability for use*, focusing on usability, intrusiveness, and workload considerations for HCPs.

Overall, participants acknowledged MyPal’s potential to enhance symptom monitoring, communication with HCPs, and holistic care delivery. They appreciated the convenience of remote reporting and the structured feedback offered by the system. Their perceptions highlight the dual potential of MyPal. On the one hand, participants recognized its value in enhancing symptom monitoring, improving patient-HCP communication, and supporting holistic care. By contrast, concerns emerged around issues of usability, digital burden, privacy, and the potential for depersonalized care. For example, participants expressed concerns about time investment, effort, and potential disruption to daily life or professional routines. Excessive notifications and complex use requirements could deter engagement, ultimately reducing the effectiveness of the intervention. Previous research has shown that high notification frequency can lead to app abandonment, with engagement declining when users perceive digital tools as intrusive [28]. Given that app-based interventions for chronic diseases report dropout rates of up to 43%, minimizing burden is critical in maximizing sustained engagement [29].

Participants also emphasized the importance of a balanced approach that integrates digital tools alongside traditional care practices to maintain the personal connection between patients and HCPs. Concerns about usability and engagement were also noted, particularly among older adults and those with limited digital literacy. While privacy and security emerged as central barriers to adoption, the findings suggest that building trust in eHealth technologies and providing user control over monitoring features are crucial to fostering long-term engagement.

Overall, the results indicate that while eHealth tools such as MyPal have significant potential to improve palliative care, success hinges on addressing concerns related to digital burden, privacy, and maintaining a sense of human connection. These insights provide a foundation for exploring how digital interventions can complement existing care practices, which is further elaborated in the subsequent sections.

### Privacy, Security, and Usability Considerations

During the early stages of the analysis process, it was evident that privacy and security concerns were among the most pressing issues raised by participants, particularly regarding facial and voice recognition features. Even when reassured about data protection measures, many participants remained unwilling to accept such monitoring functionalities. Previous research suggests that the acceptance of digital health technologies depends heavily on patient trust and transparency in security protocols [30]. Studies emphasize that both patients and HCPs must be educated on these measures to alleviate fears and improve confidence in eHealth tools [31-33]. In addition, presenting security systems in a clear and comprehensible manner has been shown to enhance user acceptance and engagement with digital health systems [34]. Beyond security, participants expressed concerns about feeling constantly monitored without control over when and how data collection occurs, a factor that has been previously identified as a barrier to adoption, as many users perceive continuous monitoring as an invasion of privacy and prefer having control over when data are recorded [35]. Addressing these concerns is critical for increasing trust and ensuring the long-term adoption of ePRO interventions. Another key issue was usability and accessibility, particularly for older adults and individuals with physical limitations. Participants noted that small font sizes, complex interfaces, and navigation difficulties could hinder engagement. Previous studies highlight how older users often struggle with digital interfaces primarily designed for younger populations, underscoring the need for age-friendly designs that accommodate diverse user capabilities [36]. To enhance accessibility, future iterations of MyPal should prioritize intuitive design, customizable interfaces, and features that ensure ease of use across all patient demographics.

### Implications

Another crucial element to consider in future eHealth palliative care solutions is patients’ and caregivers’ wishes to receive personalized feedback regarding the data they or their children submit. While ePROs augment the reporting experience, in many cases, they lack the feedback element [37], creating miscommunication issues. Therefore, the incorporation of feedback elements could optimize the user experience by making app-based reporting feel more meaningful and immediately relevant to patients, as they would receive an outcome based on their interaction with the app (eg, a message that “the doctor has seen your submission and will answer through the app in 24 hours at the latest”). In addition, by adding a co-design approach, miscommunication issues are severely limited [16]. Finally, this study did not explore any differences in perception among the participating clinical sites. Future research may address such differences to explore palliative care applications in different settings.

### Strengths

This study offers a multinational perspective by collecting data from 4 European countries, allowing a diverse range of viewpoints from different health care systems. The use of qualitative FGs provided an in-depth exploration of user perceptions, generating rich insights into the experiences of patients, caregivers, and HCPs. In addition, the inclusion of multiple stakeholder groups ensured a holistic understanding of the feasibility, benefits, and challenges of implementing digital palliative care solutions.

### Limitations

First, our sample may have consisted of participants with an interest in or familiarity with digital technology and the development of eHealth solutions, which may have introduced bias. As a result, individuals with low digital literacy, who may face greater challenges in using such tools, were underrepresented. Given that poor digital literacy can significantly impair interactions with HCPs and limit the benefits of digital health services [38], future studies should include a more diverse sample that explicitly accounts for varying levels of digital literacy.

Second, the first coding step, the inductive coding of FG data, was conducted separately at a national level by different investigators. This approach may have introduced subjective biases related to individual assumptions and analytic styles. However, to mitigate this, the aggregated initial codes were later organized into overarching categories, ensuring consistency across all participating sites and participant groups. This process helped standardize the interpretation of data and minimize potential discrepancies.

Third, the number of FGs conducted may have limited the diversity of perspectives captured. Although the study aimed to conduct at least 1 FG per user group in each country, thematic analysis suggested that additional FGs could have provided further insights. Given that palliative care needs and digital health adoption may vary across cultural and health care settings, increasing the number of FGs in future studies may help elicit a broader range of opinions and experiences.

Fourth, the validity of the FG content was another limitation. While the discussion guides were carefully designed by experts in psychology and software engineering within the MyPal project consortium, they were not directly validated by all stakeholders, such as patients and caregivers. Ensuring FG content is aligned with stakeholder-specific experiences and routine interactions with the MyPal system could improve the relevance of the findings and provide more targeted insights for future iterations of the tool.

Fifth, only limited background information was collected from FG participants, specifically age, sex, and professional role (for HCPs). Additional characteristics were excluded, such as time since diagnosis, comorbidities, current medications, or years of professional experience, primarily due to methodological constraints of the cross-national design, including heterogeneity in data availability across study sites and varying participant willingness to disclose medical or professional information. These limitations could have affected data reliability and comparability, further supporting their omission in a study primarily focused on perceptions, opinions, and needs of an eHealth solution. Future research should consider collecting more detailed personal and clinical data to better contextualize participant input. Furthermore, language barriers and the remoteness factor posed challenges in the study design. Conducting FGs in multiple languages required translation efforts, which introduced delays, and potential nuances might have been lost in translation. In addition, coordinating a multilingual consortium created logistical complexities, as experts from different countries had to communicate in a nonnative language while designing FG materials. This process increased the time and effort needed for study preparation and may have introduced subtle inconsistencies in content delivery. Finally, although no major thematic differences emerged across countries, the possibility of subtle cultural or systemic variations being underrepresented due to data aggregation cannot be entirely ruled out. Future research could address this by incorporating bilingual analysts or using artificial intelligence–driven translation validation methods to enhance accuracy and efficiency.

### Comparison to Prior Work

A key insight from this study was the value participants placed on maintaining the physician-patient relationship through MyPal. While digital tools were seen as beneficial for enhancing communication, there was skepticism regarding features that could lead to a depersonalized experience. Previous studies have shown that maintaining “real-person” contact is crucial for patient satisfaction, and the acceptability of mobile health consultation apps remains limited due to concerns about impersonal interactions [39]. To address this, future eHealth solutions should integrate the participatory design paradigm to align digital interventions with patients’ and caregivers’ needs, ensuring they complement rather than replace in-person interactions [15,34]. In addition, participants emphasized the need for clearer guidelines on the use of MyPal, particularly regarding when to report symptoms via the app versus seeking direct medical attention. A previous study indicated that digital health interventions must clearly define user roles and expected outcomes to ensure effective engagement and adherence [40]. Without such clarity, users may struggle to understand how the tool integrates into their health care, potentially reducing its effectiveness.

### Conclusions

This study provides important insights into the feasibility, benefits, and challenges of implementing ePRO-based digital interventions in palliative care. While MyPal was perceived as a valuable tool for symptom monitoring and communication, concerns regarding usability, data security, and clinical integration must be addressed for successful adoption. Findings reinforce the need for patient-centered eHealth interventions that complement, rather than replace, direct interactions in palliative care. The participatory design approach used in this study played a crucial role in ensuring meaningful stakeholder involvement, fostering patient empowerment, and promoting long-term engagement with the technology. Future work should focus on refining digital solutions based on user feedback to optimize engagement and clinical impact.

## Appendix

Multimedia Appendix 1 Adult patient vignettes, pediatric patient and parent vignettes, sample slides from the focus group vignette of pediatric patients and their parents, health care professional vignette, and sample slides from the focus group vignette of health care professionals.

Multimedia Appendix 2 Discussion guide for the focus group of adult patients.

Multimedia Appendix 3 Training workshop screenshots.

## Abbreviations

BRNO: Fakultní nemocnice Brno
CERTH: Centre for Research and Technology Hellas
CLL: chronic lymphocytic leukemia
ePRO: electronic patient-reported outcome
FG: focus group
HCP: health care professional
HM: hematological malignancy
INAB: Institute of Applied Biosciences
MDS: myelodysplastic syndrome
MHH: Medizinische Hochschule Hannover
PAGNI: Panepistimiako Geniko Nosokomeio Irakleiou
QoL: quality of life
USAAR: Universität des Saarlandes
USR: Università Vita-Salute San Raffaele

## Data Availability

The datasets generated or analyzed during this study are not publicly available due to privacy and General Data Protection Regulation requirements. However, they are available from the corresponding author on reasonable request.
