# Supplementary material for: Perceptions and Needs of Stakeholders Regarding MyPal Project’s Electronic Patient-Reported Outcome App: Cross-Sectional Qualitative Focus Group Study
Source: JMIR Cancer. 2025 Aug 13;11:e57388. doi: 10.2196/57388 (PMC12391845; doi:10.2196/57388)

## Appendix A

### Adult Patient’s Vignettes

The vignette introduces the main character, a patient called Mr Leonard Jones and his journey of managing Chronic Lymphocytic Leukemia. The scenario starts with the enrollment of the patient into the study. In this first episode (Day 0) he signs an informed consent, which includes permission for smartphone sensors to record in the background. He also receives the MyPal App, a smartphone application as well as a smart wristband which can monitor physical activity and vital signs. Finally, he completes the entry questionnaires which shape the personalization aspects of the MyPal intervention. At Day 7, Mr Jones receives a motivational message which aims to keep him engaged to the MyPal system. The next episode (Day 14) takes place after two weeks, when Mr Jones receives a notification to fill in the 1^st^ symptom questionnaire via which he reports some fatigue. Mr Jone’s fatigue is also becoming evident through a reduction in his physical activity. In the next episode (Day 18), Mr Jones searches for information in the personalized medical information search. He finds out that symptoms such as fatigue combined with a rash and swollen lymph nodes can signal the progression of CLL. At Day 22 Mr Jones develops rashes which he reports by using the spontaneous reporting functionality of MyPal app. He uploads a photograph and marks all his symptoms including swollen lymph nodes on a list. As all indicators (spontaneous reporting, symptom questionnaires and wristband data) demonstrate that CLL has progressed, MyPal issues an alert. The clinician contacts Mr Jones and asks him to undergo blood tests. At Day 24, After the blood results verify this, Drug D is prescribed to Mr Jones. He checks for drug interactions and inserts notifications to receive notifications on the MyPal app. Mr Jones continues to receive motivational messages and self-report questionnaires on physical and psycho-emotional symptoms to complete. Mr Jones keeps experiencing diarrhea so at Day 42 he reports it via MyPal app and his clinician is notified. As the diarrhea insists, he begins to experience distress. This is evident in his facial expressions and tone of voice. Due to the distress the smartphone sensors have picked up and the spontaneous reporting of a symptom Mr Jones Jones has performed, the clinician is alerted and calls to reassure him.

### Sample Slides from the Focus Group User Vignette of Adult Patients


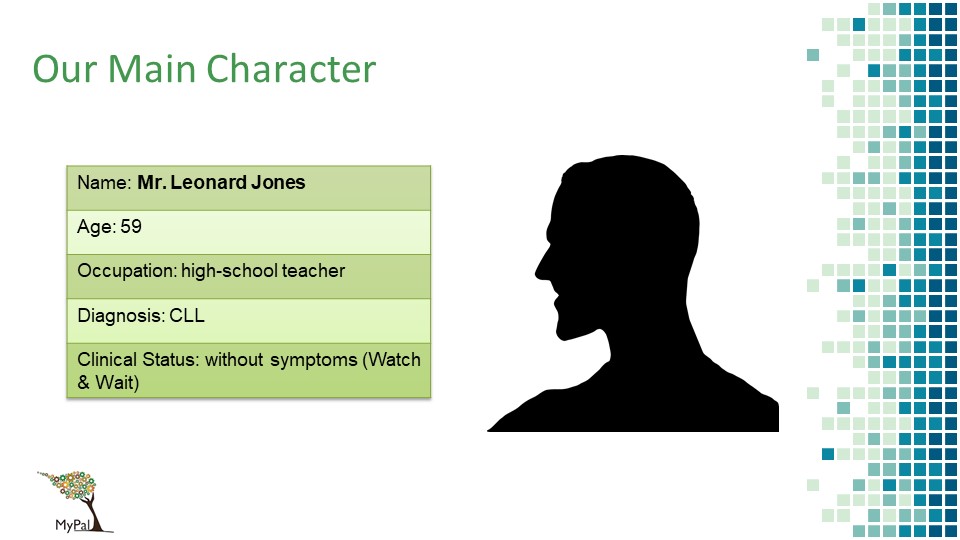


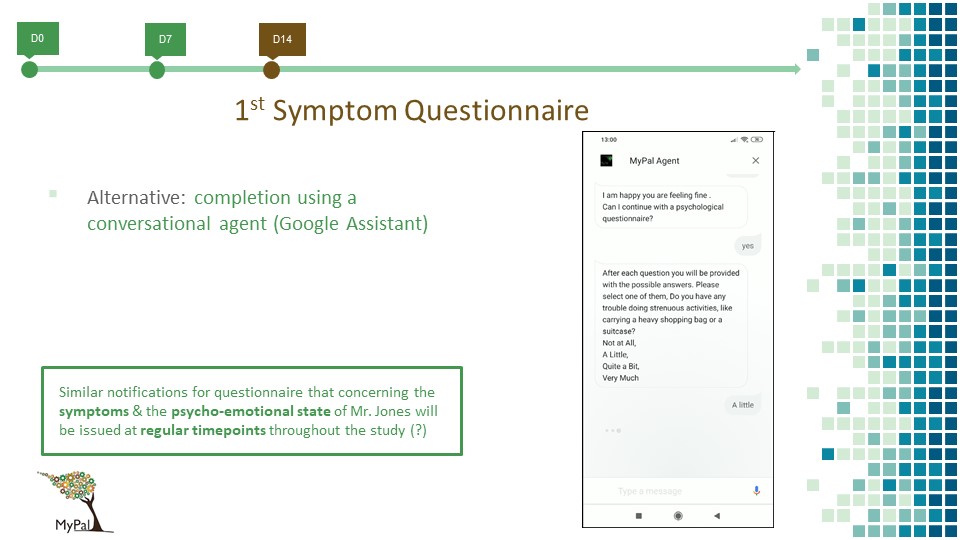


### Pediatric Patients and Parents Vignette

The vignette presented introduces two main characters, the patient George and his mother Julia and their journey of managing metastatic sarcoma. The scenario starts with the enrollment of the patient into the study. In this first episode (Day 0) he signs an informed consent, which includes permission for smartphone sensors to record in the background. He also receives the MyPal App, a smartphone application with many features as well as the MyPal game. This will include the runner game, the augmented reality activation game and a pure questionnaire module. Finally, he completes the entry questionnaires which shape the personalization aspects of the MyPal intervention. At Day 14, George is playing the game and Julia receives an SMS that encourages her to ask George whether he is experiencing a symptom. The next episode (Day 20) takes place after a week, when George while playing the game, reports some fatigue. At Day 35 George is in pain and reports it via MyPal. His fatigue is also becoming evident through a reduction in his physical activity. His clinician is notified and asks Julia to bring her son in for a visit. At Day 39 the checkup is complete and results indicate that George need a new line of chemotherapy. George can use MyPal to connect with schoolfriends while he is in the hospital. At Day 40, distress is detected through the help of smartphone sensors facial expressions and tone of voice as well as a self-report questionnaire indicating the experience of distress. The clinician is notified and calls Julia to support her while George gets a motivational message via the game and starts feeling somewhat better. In the meantime, Julia looks through the drug interaction feature of MyPal and sets up notifications for receiving treatment. In the next episode (Day 41), Julia and George search for information in the personalized medical information search as they are worried about side effects. They find out that different treatment drugs have different side effects but these can often be prevented or treated early. At Day 43, George develops diarrhea, an adverse event and registers this via MyPal app so that the clinician is notified. At Day 48, due to early intervention diarrhea stops. George and Julia have completed a number of physical and psycho-emotional symptom questionnaires and received several motivational messages on their smartphones. At the end of this line of treatment (Day 304) George is feeling fine and still playing the MyPal game. Julia has experienced the benefits of Mypal and still using the app and all its functionalities.

### Sample Slides from the Focus Group Vignette of pediatric patients and their parents


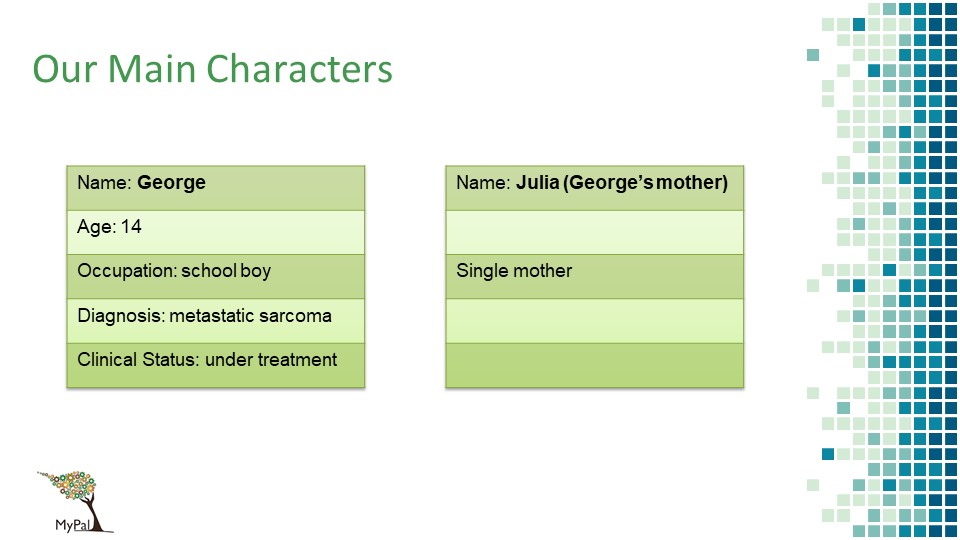


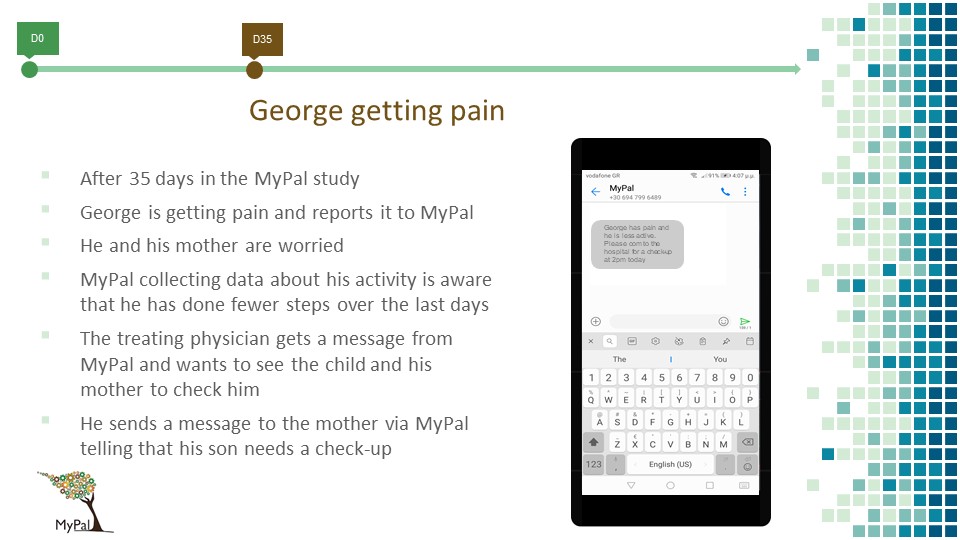


### Healthcare Professionals Vignette

The vignette presented introduces two main characters, the patient Mr Leonard Jones and the clinician Dr. Cecilia Salmann and their journey of managing Chronic Lymphocytic Leukemia. The scenario starts with the enrollment of the patient into the study. In this first episode (Day 0) he signs an informed consent, which includes permission for smartphone sensors to record in the background. He also receives the MyPal App, a smartphone application as well as a smart wristband which can monitor physical activity and vital signs. Finally, he completes the entry questionnaires which shape the personalization aspects of the MyPal intervention. Equivalently, Dr Cecilia also receives a smartphone/tablet app and credentials in order to be able to log on to the clinicians’ website and she is trained on how to use them. The next episode (Day 14) takes place after two weeks, when Mr Jones receives a notification to fill in the 1^st^ symptom questionnaire via which he reports some fatigue. The collected patients’ data (reported & sensed data as well as the medical history) is made available to Dr. Salmann via the clinicians’ website. In the next episode (Day 22), Mr Jones searches for information in the personalized medical information search, where Dr Salmann and her colleagues had previously spent some time inserting validated medical content. Mr Jone’s fatigue is also becoming evident through a reduction in his physical activity. At Day 36 Mr Jones develops rashes which he reports by using the spontaneous reporting functionality of MyPal app. He uploads a photograph and marks all his symptoms including swollen lymph nodes on a list. Previously Dr. Salmann had studied the analytics webpage and had designed a spontaneous symptom reporting form that highlighted ‘stealth symptoms’ such as swollen lymph nodes. In the next episode, MyPal issues an alert as all indicators (spontaneous reporting, symptom questionnaires and wristband data) demonstrate that CLL has progressed. Dr Cecilia contacts Mr Jones and asks him to undergo blood tests. At Day 38, After the blood results verify this, Drug D is prescribed to Mr Jones. At Day 45 Mr Jones develops diarrhea, an adverse event, due to which he experiences distress. This is evident in his facial expressions and tone of voice. Due to the distress the smartphone sensors have picked up and the spontaneous reporting of a symptom Mr Jones Jones has performed, Dr Salmann is alerted and calls to reassure him. During the phone call, she discusses the pros and cons of treatment, with the help of a conversation guide offered by MyPal, taking into account Mr Jones’s answers to a screener treatment belief questionnaire.

### Sample Slides from the Focus Group Vignette of HCPs


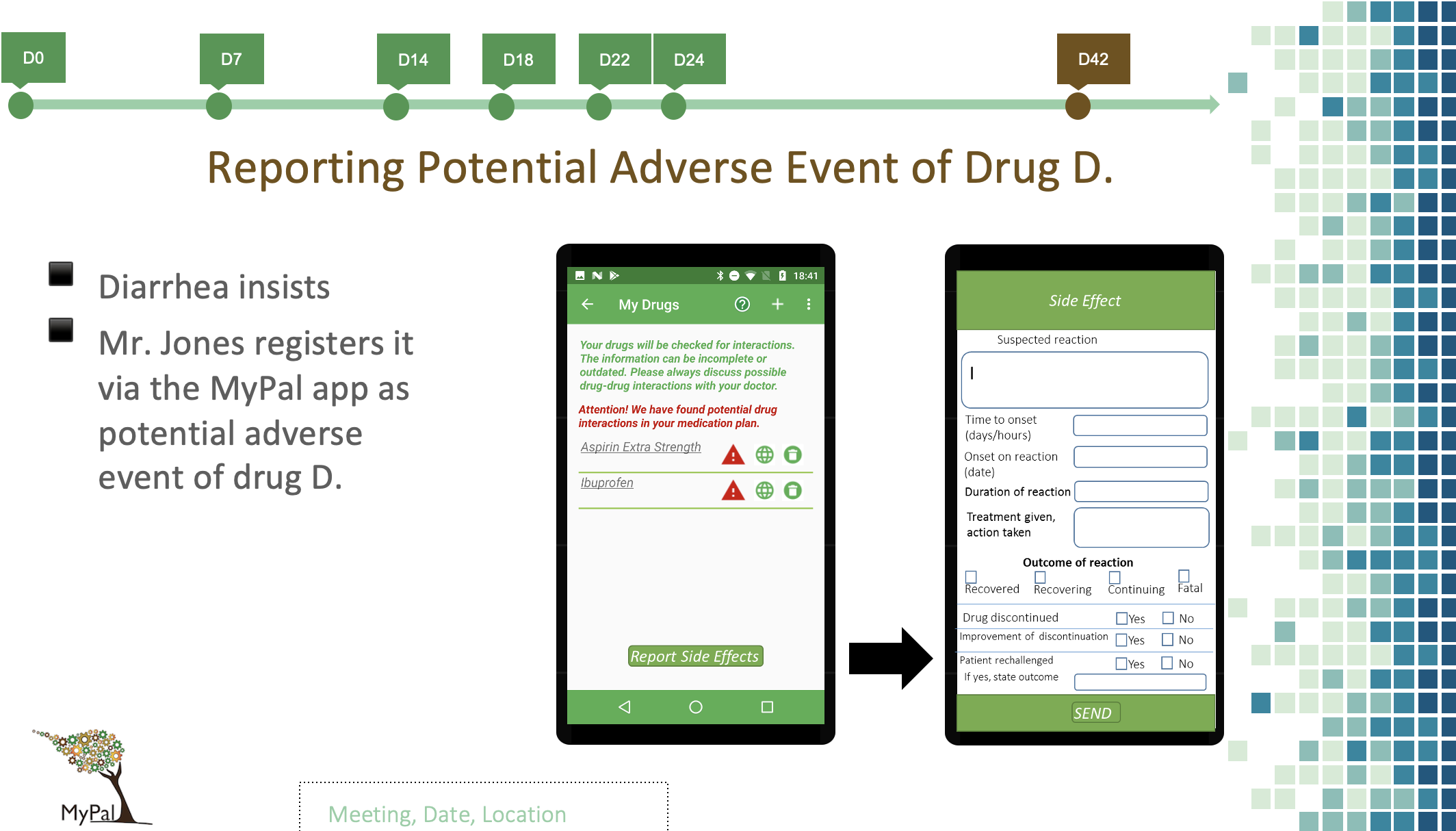


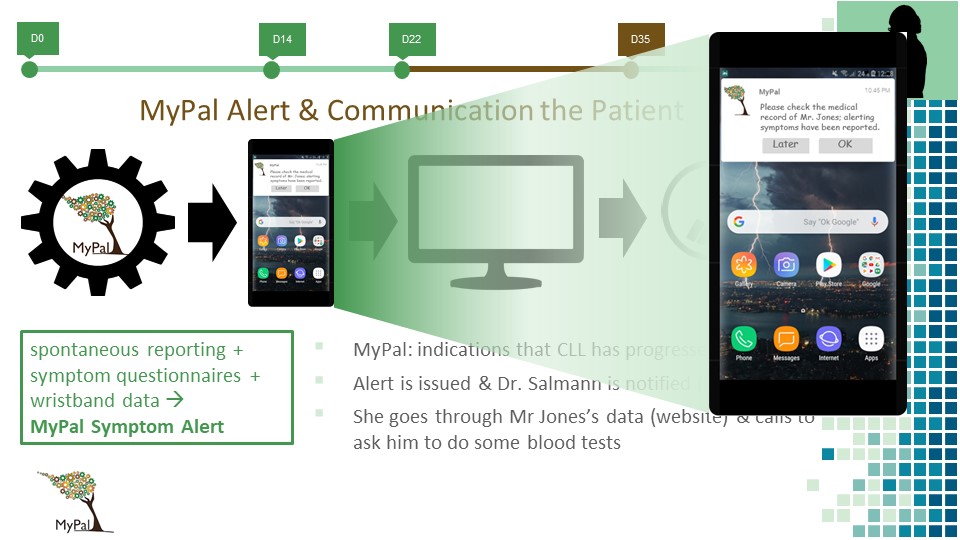

Supplement: Multimedia Appendix 1 [file cancer_v11i1e57388_app1.docx]
