# Supplementary material for: Perceptions and Needs of Stakeholders Regarding MyPal Project’s Electronic Patient-Reported Outcome App: Cross-Sectional Qualitative Focus Group Study
Source: JMIR Cancer. 2025 Aug 13;11:e57388. doi: 10.2196/57388 (PMC12391845; doi:10.2196/57388)
Supplement: Multimedia Appendix 2 [file cancer_v11i1e57388_app2.docx]

## Appendix B

### Discussion Guide for Focus Group of Adult Patients

Textbox 1. The caption/title is placed here in a sentence format (capitalization of every word is unnecessary).

| Hello, I am XX from XX…, the institution in charge of the study which brings us here together today.  As you know, we are going to talk about MyPal project and we will be asking you your opinion on several matters so that we can plan according to patients’ needs.  But before we begin our discussion on this subject, may I ask each of you to introduce him / herself.  **INTRODUCTION**  The objective in this section is to introduce a) MyPal project as a whole b) the structure of today’s meeting.  MyPal project is……  Today we thought we would present MyPal to you by presenting the story of a CLL patient, Mr Jones, and his journey through his illness with MyPal.  Storyline: Mr Jones is a XX year old man who lives XX and was diagnosed with CLL  Let’s see the story again from the beginning….  **DISCUSSION**  **1.**  At day 0 we see Mr Jones consenting to receive a screener questionnaire to complete sometime in the future. Essentially what that does is help create a profile of Mr Jones Creating a profile means that all information or messages sent in the future to Mr Jones are relevant to him; in other words they are personalized.   - Spontaneous reactions. - What do think about that? - How important do you judge this step to be? - How would you feel if you had the option to offer information about yourself in order to personalize the content of a system like MyPal?   **2.** After that, we see Mr Jones receiving a wrist band which will transmit information about his physical status. For example, it could monitor his physical activity or his heart rate.   - Spontaneous reactions. - How important do you feel it is that this information is communicated to your doctor via MyPal? - What do you think about the idea of monitoring your physical function remotely?   <<SHOW WRISTBAND (if available)>>   - How would it make you feel to wear a wrist band for most of the day every day? (e.g. safe? tedious?) - What kind of problems do you foresee? - How do you think it would contribute to your overall care? - What other aspects of your physical status do you think could be monitored to help you manage your condition more effectively?   **3.** Mr Jones also gave his permission for MyPal to record images or sound via his mobile. Later on, the data was used to diagnose distress in Mr Jones and his clinician was notified.   - Spontaneous reactions. - How would you feel about giving permission to be recorded in the context of MyPal? (e.g. worried/not bothered) - How helpful do you think such as function is? - What are some of the reasons for / against going through with this?   **4.** Later on, Mr Jones receives a notification to complete a symptom questionnaire. This could concern physical or psychoemotional symptoms.   - Spontaneous reactions - How helpful do you judge a notification to be? Where would it best for you to receive? - What do you see the function of the symptom questionnaire to be in self-observation/communication with physician/overall care management?   <<SHOW ESAS QUESTIONNAIRE>>   - How do you find the format of the questionnaire as it is presented?   Question arrangement/ questions order/ sliders   - What do you like/ dislike? - How can it be improved?   **5.** Mr Jones goes through a smooth ‘watch and wait’ during which time he receives notifications i.e. to continue symptom reporting.   - How helpful do you judge such a notification to be? - What are some of the benefits of receiving personalized notifications? - How effective do you think personalized notifications are? - Where would like to receive notifications? (viber, whatsup, facebook messenger) - When would it best to receive them? (time of day)   **6.** Mr Jones starts feeling fatigue and searches for info since he thinks this might indicate disease progression. The information that he finds via MyPal is personalized meaning information relevant to him as a CLL patient.   - Spontaneous reactions - How helpful do you think such a feature is? - How would you feel given the opportunity to access information tailored for you as a CLL patient?   <<SHOW SEARCH OPTION>>   - How do you judge this feature? - What is the strongest point of this feature? - How often would you use it? - How can it be improved?   **7.** Mr Jones reports he has developed a rash. In addition, the wrist band indicates low physical activity, therefore his clinician is alerted via MyPal. Mr X is referred for bloodwork.   - Spontaneous reactions - What do you think of the fact that Mr Jones can report health related events via MyPal? - How would having that communication option make you feel? - In what ways might this be helpful/not helpful? - Were your expectations with regards to clinician communication in this context met? - How could this feature be made better?   **8.** Mr X is now on medication and vigilant for adverse events. When he experiences an adverse event he uses the system to communicate with the clinician. The MyPal system senses the distress that Mr X experiences via his voice and facial expressions and the clinician is notified.   - Spontaneous reactions - What do you think of the fact that Mr X can report adverse events via MyPal? - Would you use this feature? - How would having this option make you feel? (safe/anxious) - How does this feature respond to your need of effectively communicating adverse event reporting?   <<SHOW FEATURE>>   - How effective do you think MyPal in sensing distress? - How would you feel about MyPal alerting your physician after ‘reading’ your distress? - In what ways might this be /not helpful? - What do you see the function of this feature to be in self-observation/communication with physician/overall care management? |
| --- |

### Discussion Guide for Focus Group of Children with Cancer and their Parents

Textbox 1. The caption/title is placed here in a sentence format (capitalization of every word is unnecessary).

| Hello, I am XX from XX…, the research institution in charge of the study which brings us here together today.  As you know, we are going to talk about MyPal project and we will be asking you your opinion on several matters so that we can plan according to according to yours and your children’s needs.  **INTRODUCTION**  **The objective in this section is to introduce a) MyPal project as a whole b) the structure of today’s meeting.**  MyPal project is……  Today we thought we would present MyPal to you by presenting the story of a George a 14 year old patient, and Julia his mother, and their cancer journey with MyPal.  Storyline: George was diagnosed with…..  **DISCUSSION**  Let’s see the story again from the beginning….  **1.**  At day 0 we see George receiving the MyPal game on his tablet and Julia receiving the MyPal app on her smartphone. Through the game George is able to stay in touch with his healthcare team by recording at several instances, either spontaneously or prompted, his symptoms, feelings and the quality of his life. Julia also has the opportunity to record George’s symptoms as well as record her own emotional state, the quality of her life and her satisfaction with care.   - Spontaneous reactions. - What do think about the idea of eliciting information about children’s physical and emotional sensations through a game? - How important do you think it is for parents are involved in this effort by offering their own insight?   **2.** At day 0 both George and Julia received questionnaires to complete. The reason for this is that the MyPal system is trying to build a profile on both of them. This means that all the information they receive from then on will be personalized. For example, George might see his favorite avatar in the game and Julia might receive motivational or rewarding messages when using the MyPal app regularly.   - Spontaneous reactions. - How important do you feel that personalization is for children when using the MyPal game? - How important do you feel that personalization is for adults when using the MyPal app?   **3.** At day 20 George reports a mild or not intense symptom through MyPal game. George’s and Julia’s mind is put to rest, right after reporting, with regards to the symptom’s importance or relation to cancer.   - Spontaneous reactions. - How would it make your child feel to receive a response through MyPal game right after reporting? (e.g. safe? relief?) - How would it make you feel to receive a response through MyPal game right after reporting? (e.g. safe? relief?) - How helpful do you think such as function is? - What kind of problems do you foresee? - How do you think it would contribute to your child’s care?   **4.** At day 35 both George and Julia report a visible symptom through MyPal. Moreover, the MyPal system detects, with the help of sensors, a drop in George’s physical activity. Therefore, they are called into the hospital for a series of blood and imaging tests.   - Spontaneous reactions - What do you see the function of the symptom reporting to be in observation or self-observation? In communication with physician? In overall care management? - How helpful do you perceive sensors to be? (reliable? effective?)   **5.** At day 39 results are in and George needs treatment. Julia is informed.   - How smart do you perceive the MyPal system to be (in this scenario? - What is the role you can forsee for the MyPal system?   **6.** At day 48 both George and Julia report distress. MyPal system alerts the physician who calls Julia. Through the MyPal game George’s avatar makes him feel better.   - Spontaneous reactions - How would you feel about the MyPal system alerting your physician after sensing your distress? - In what ways might this be helpful? - What do you see the function of this feature to be in self-observation/communication with physician/overall management? - What do you think of the fact that George can report feelings through the MyPal game? - How helpful do you think such a feature is? - How would you feel if your child were given this opportunity? - How do you feel this feature would respond to your child’s emotional needs?   **7.** How do you think the MyPal game has helped George?   - Spontaneous reactions. - Probe:   - Physically? How?   - Psychologically? How?   **8.** What do you think about having a digital tool like MyPal to assist you in managing your child’s condition?   - Spontaneous reactions - Probe:   What is your favorite feature/ least favorite feature?  What do you think is the most useful/least useful feature?  If you had the option of choosing (or adding) features, which ones would you choose?  Which ones do you find the most novel?  **9.**  Would you recommend it to other parents/ children? |
| --- |

### Discussion Guide for Focus Group of HCP

Textbox 1. The caption/title is placed here in a sentence format (capitalization of every word is unnecessary).

| Hello, I am XX from XX…, the institution in charge of the study which brings us here together today.  As you know, we are going to talk about MyPal project and we will be asking you your opinion on several matters so that we can plan according to patients’ needs.  But before we begin our discussion on this subject, may I ask each of you to introduce him / herself.  **INTRODUCTION**  **The objective in this section is to introduce a) MyPal project as a whole b) the structure of today’s meeting.**  **MyPal project is……**  Today we thought we would present MyPal to you by presenting the story of a Mr Jones and Dr Salmann, and their journey of managing his illness with MyPal.  **Storyline: …….**  Let’s see the story again from the beginning….  **DISCUSSION**  **1.**  At day 0 we see Mr Jones consenting to take part in MyPal. This includes receiving the MyPal app, a smart wristband, an entry questionnaire. Furthermore, Mr Jones gives his permission for smartphone sensors (including camera, microphone) to record in the background. The goal is to personalize the MyPal intervention for each patient.   - Spontaneous reactions. - How important do you judge enrollment to be? - How do you think your patients would react to personalized capabilities offered by a system like MyPal? - What do you think about that?   **2** Mr Jones receives a wrist band which will transmit information about his physical status. For example, it could monitor his physical activity or his heart rate.   - Spontaneous reactions. - How important do you feel it is that this information is communicated to the clinician via MyPal? - What do you think about the idea of monitoring your patient’s physical function remotely? - How do you think it would contribute to your patient’s overall care? - What other aspects of patients’ physical status do you think could be monitored to help you manage their condition more effectively? and how?   **3** Mr Jones also gave his permission for MyPal to record images or sound via his mobile. Later on, the data is used to diagnose distress and the clinician is notified.   - Spontaneous reactions. - How helpful do you think such a function is? - What are some of the reasons for / against going through with this? - How do you think your patients would react to this MyPal feature? - Would you trust the automatic distress detection feature of MyPal?   **4** When registering in the MyPal study, Dr Cecilia receives a smartphone/tablet app and credentials for clinicians’ website. She is trained on how to use them.   - Spontaneous reactions. - How important do you judge this step to be? - How helpful do you think training would be in this context? - What are some of the problems you foresee? - What are some of the reasons for /against this feature of MyPal? - How do you judge MyPal΄s capability of connecting with patients?   **5** Later on, Mr Jones receives a notification to complete a symptom questionnaire. This could concern physical or psychoemotional symptoms. He reports some fatigue.   - Spontaneous reactions - What do you see the function of the symptom questionnaire to be in:   Observation/ monitoring  Communication with physician  Overall care management  How do you anticipate that symptom observation/monitoring within MyPal can benefit the patients?  **6** Dr Cecilia X has access to all collected patient data; (reported, sensed as well as medical history) through the clinicians’ website.   - Spontaneous reactions - How do you judge this capability? - How often would you use it? - Where would like this information to be available (i.e. smartphone)?   **7** Mr Jones starts feeling fatigue and searches for info since he thinks this might indicate disease progression. The information that he finds via MyPal is personalized namely information relevant to him as a CLL patient.   - Spontaneous reactions - How helpful do you think such a feature is? - What do you think about the idea that patients have the opportunity to access information tailored to their needs?   **8.** Dr Cecilia and her colleagues had spent some time to insert validated medical content (e.g. recent high-quality scientific articles) in the MyPal database.   - Spontaneous reactions - What are your expectations with regards to helping advance patients’ health literacy? - Would you say that this would enhance their ability to manage their illness better? - How could this feature be made better? - How much time would you be willing to spend for such a task?   **9** Mr Jones reports he has developed a rash. In addition, the wrist band indicates low physical activity, therefore his clinician is alerted via MyPal. Mr Jones is referred for bloodwork.   - Spontaneous reactions - Which is your preferred channel for communicating with your patients? - What do you think of the fact that Mr Jones can report health related events via MyPal? - How would having that communication option open make you feel? - In what ways might this be helpful/not helpful? - Are your expectations with regards to clinician-patient communication in this context met? - How could this feature be made better?   **10** Dr Cecilia can access aggregate data from all study participants. She discovers that study participants tend to overlook swollen lymphnodes in spontaneous reporting. She decides to design a spontaneous symptom reporting form that highlights stealth symptoms such as lymphnodes.   - Spontaneous reactions - What do you think of the fact that clinicians can look at their patients’ data as a group? - How do you judge the clinician’s opportunity to elicit the information he /she needs in a structured way? Is it something that you would be interested in using? - How could this feature be made better? - How would like to exploit MyPal trends and needs discovered by MyPal in the aggregated patient data?   **11** Mr Jones is now on medication and vigilant for adverse events. When he experiences an adverse event he uses the system to communicate with the clinician. The MyPal system senses the distress that Mr Jones experiences via his voice and facial expressions and the clinician is notified.   - Spontaneous reactions - What do you think of the fact that Mr Jones can spontaneously report adverse events via MyPal? - According to your opinion, how does this feature respond to patients’ needs of effectively communicating adverse event reporting? - What is in your opinion the most effective way for spontaneous symptom reporting? Via a structured for or via free-text? - In what ways might this be /not helpful? - What do you see the function of this feature to be in:   Observation/ monitoring  Patient -Physician communication  Overall care management  **12** Mr Jones indicates that he has some doubts about his treatment via a screener questionnaire. Dr Salmann takes his answers into account and uses the discussion guide and her own expertise to talk to him about the pros and cons of treatment.   - What do you judge the role of the screener questionnaire to be? - How useful do you feel it would be for a HCP to have this information in a consultation? - How do you judge the clinician’s access to a tool such as a structured discussion guide to help him manage a challenging conversation? - Does this tool match your expectations on doctor-patient communication tools?     **13** How do you think MyPal has helped Mr Jones?   - Spontaneous reactions. - Probe:   Physically? How?  Psychologically? How?  **14** What do you think about having a digital tool like MyPal to assist you in managing CLL?   - Spontaneous reactions - Probe: - What is your favorite feature/ least favorite feature? - What do you think is the most useful/least useful feature? - What would rather use app or web? In which instances? - If you had the option of choosing features, which ones would you choose? - Which ones do you find the most novel? |
| --- |
