# Supplementary figures and images for: Perceptions and Needs of Stakeholders Regarding MyPal Project’s Electronic Patient-Reported Outcome App: Cross-Sectional Qualitative Focus Group Study
Source: JMIR Cancer. 2025 Aug 13;11:e57388. doi: 10.2196/57388 (PMC12391845; doi:10.2196/57388)

## Appendix C

### Training Workshop Screenshots


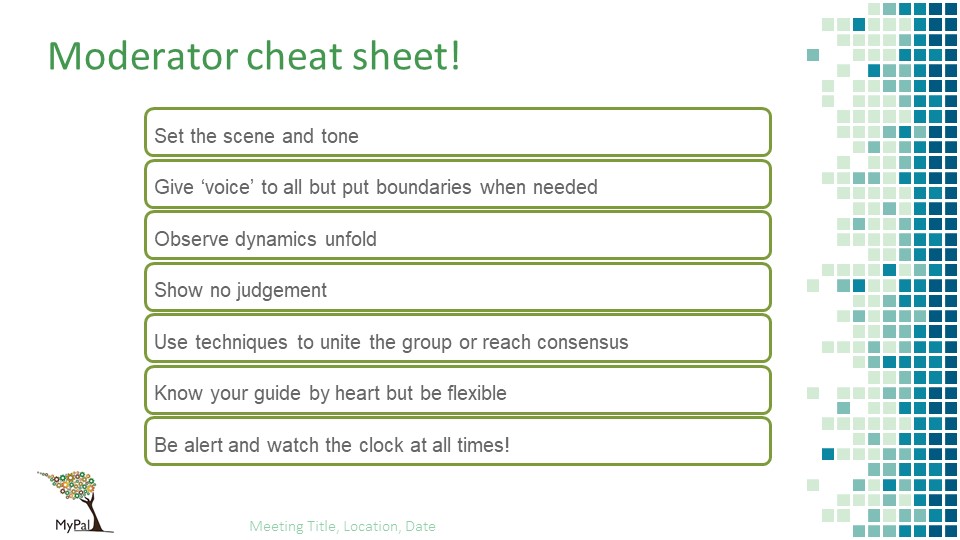


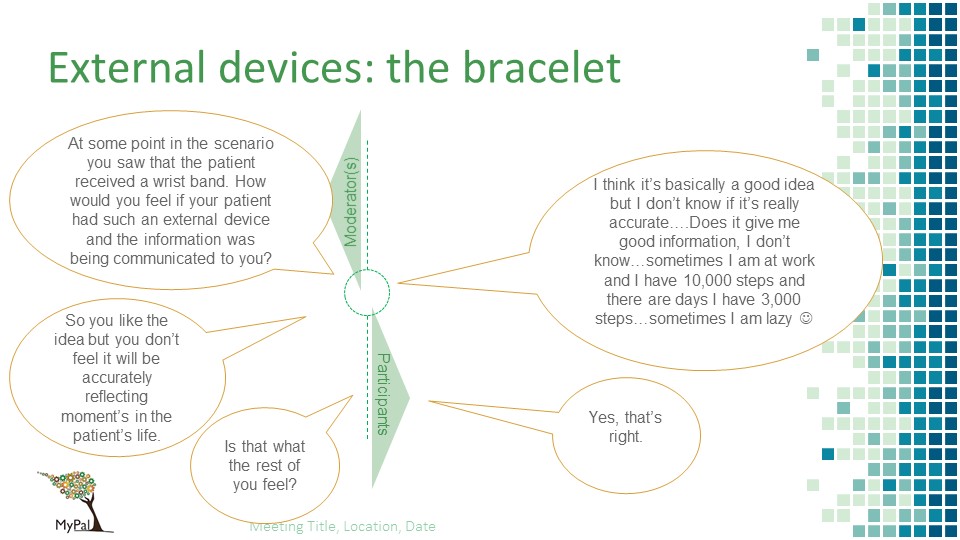


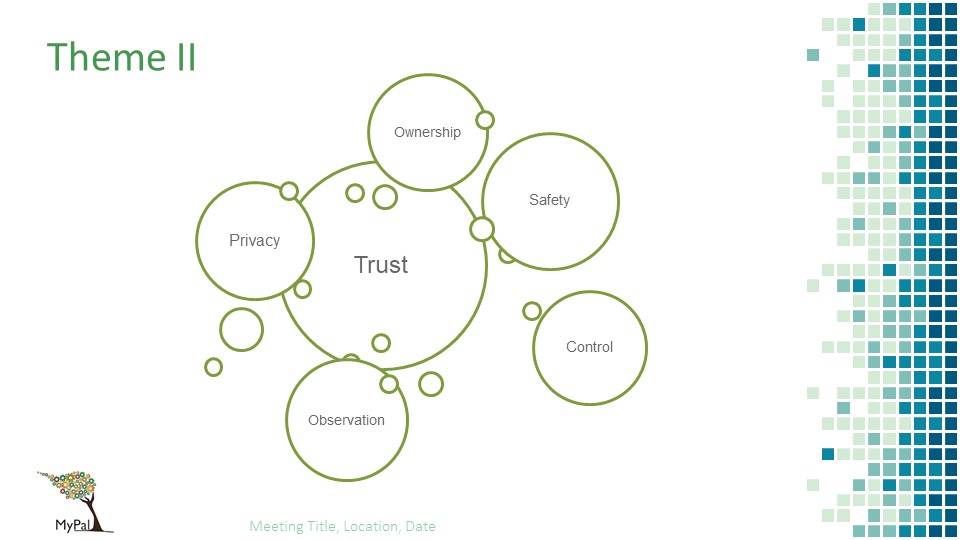


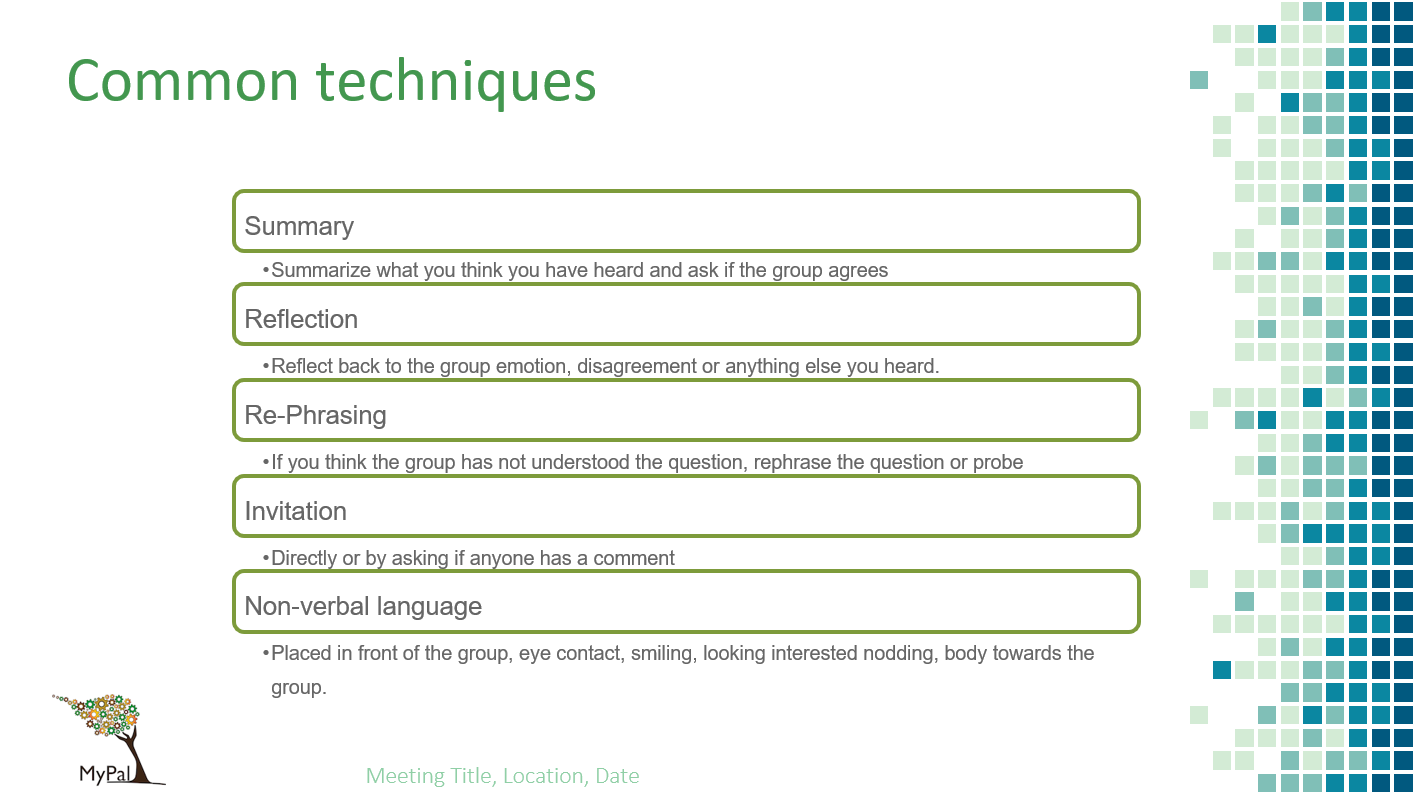


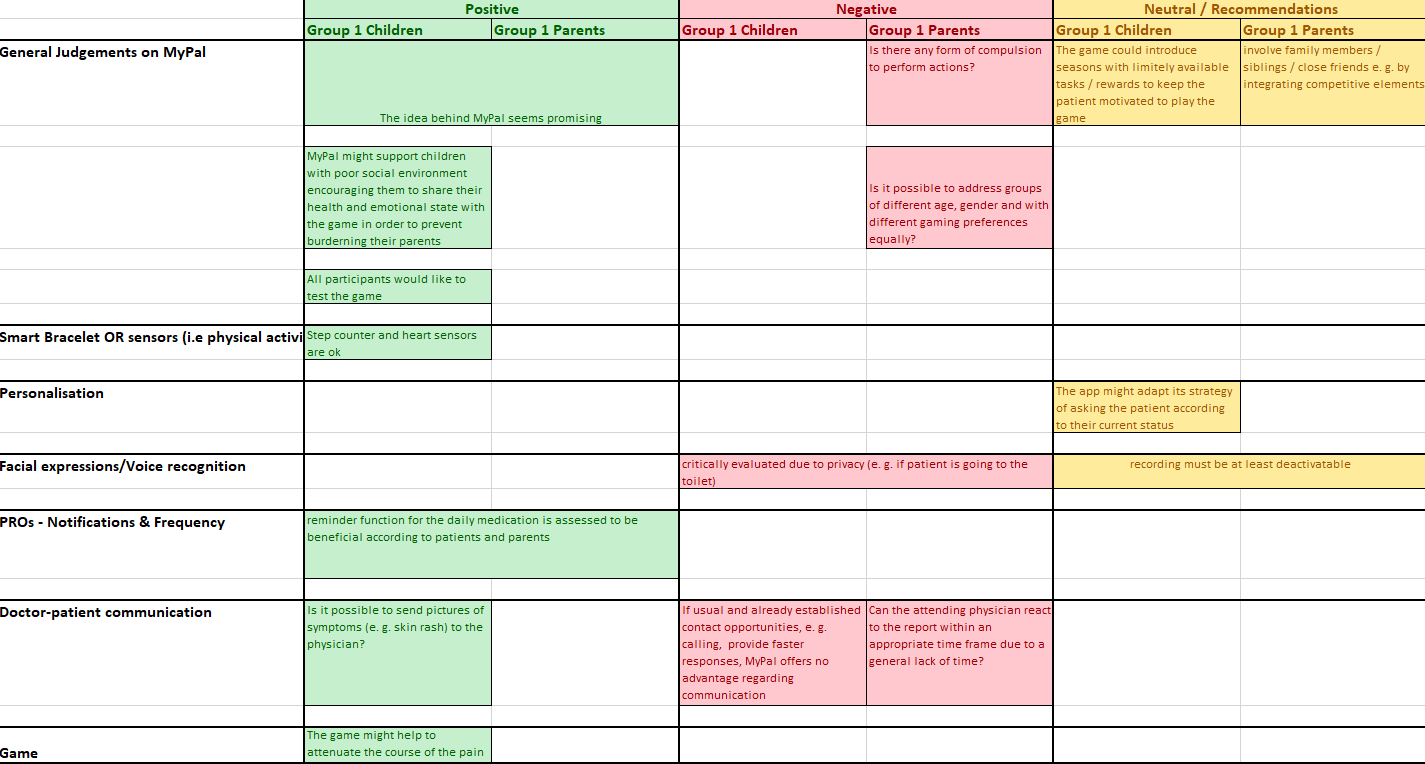


Figure 3. Coding Book for Focus Group Organizers

Supplement: Multimedia Appendix 3 [file cancer_v11i1e57388_app3.docx]
